# Supplementary material for: Molecular and Serological Detection of Piroplasms in Horses from Nigeria
Source: Pathogens. 2021 Apr 23;10(5):508. doi: 10.3390/pathogens10050508 (PMC8146079; doi:10.3390/pathogens10050508)
Supplement: Supplementary file 1 [file pathogens-10-00508-s001.zip › pathogens-1173654-supplementary.pdf]

Supplementary Table 1. Hematology, molecular, and serological data of horse samples collected in Nigeria.

	Hematology			nPCR			cELISA	
Horse ID	RBC **($\times10^6$/μL)**	PCV **(%)**	Hb **(g/dL)**	*T. equi*	*T. haneyi*	*B. caballi*	*T. equi*	*B. caballi*
2	7.71	34.6	13.5	+	-	-	+	-
4	6.41	28.7	10.6	-	-	-	+	-
6	6.62	33.7	12.4	+	-	-	+	-
7	3.72	19.2	7.1	+	-	-	+	-
8	7.02	22.9	12.4	-	-	-	-	-
9	6.67	34	12.1	-	-	-	+	-
10	2.78	10.7	4.4	-	-	-	+	-
12	6.19	28.9	11	-	-	-	+	-
14	5.79	27.7	10.2	+	-	-	-	-
15	6.18	27.2	10.1	+	-	-	+	-
16	6.55	30.2	10.8	+	-	-	+	-
17	7.62	33.1	12.4	+	-	-	-	-
18	6.94	35	12	-	-	-	+	-
20	5.79	26.6	9.9	+	-	-	+	-
26	10.72	58.6	19.7	+	-	-	+	-
29	5.91	26.2	9.7	+	-	-	+	-
Z-1	6.44	30.6	10.2	+	-	+	+	-
Z-2	6.49	32.6	10.8	+	-	-	+	-
Z-3	6.3	31.9	10.3	-	-	-	+	-
Z-4	5.82	26.6	9	-	-	-	+	-
Z-5	5.7	28.4	9.3	-	-	-	+	-
Z-6	6.25	29.8	10.4	-	-	-	+	-
Z-7	5.25	27.4	9	+	-	-	+	-
Z-8	5.7	26.6	8.9	+	-	-	+	-
Z-9	7.5	42.1	14.1	+	-	-	+	-
Z-10	9.2	45.4	16.4	-	-	-	+	-
Z-11	4.81	27.3	8.6	+	-	-	+	-
Z-12	2.15	11.1	3.9	-	-	-	+	-
Z-13	6.74	30.6	10.5	-	-	-	+	-
Z-14	7.86	37.8	12.6	-	-	-	-	-
Z-15	7.18	37	11.4	-	-	-	+	-
Z-16	6.41	31.8	10.2	-	-	-	+	-
Z-17	6.39	31.4	10.8	-	-	-	+	-
Z-18	6.86	36.6	11.3	+	-	-	+	-
Z-19	7.45	36.4	11.7	-	-	-	+	-
Z-20	7	34.8	11.2	+	-	-	+	-
Z-21	7.89	40.6	12.9	-	-	-	+	-
Z-22	6.81	37	11.3	+	-	-	+	-
Z-23	7.64	35.8	11.8	-	-	-	+	-
Z-24	5.38	28.2	8.8	+	-	-	+	-

Z-25	5.88	30.8	9.5	-	-	-	+	-
Z-26	5.77	31.6	9.5	-	-	-	-	-
Z-27	7.5	35.8	11.7	-	-	-	+	-
Z-28	6.65	34.1	11	-	-	-	+	-
Z-29	6.71	35	11.8	-	-	-	+	-
Z-30	6.52	30.7	10.7	-	-	-	+	-
Z-31	7.63	38.2	13	-	-	-	-	-
Z-32	6.94	34.1	11.8	-	-	-	+	-
Z-33	6.33	35.4	11.1	-	-	-	+	-
Z-34	7.09	37.4	11.7	-	-	-	+	-
Z-35	5.56	29.8	9.3	-	-	-	+	-
Z-36	5.95	33.1	10.3	-	-	-	+	-
Z-37	8.54	43.4	14.9	-	-	-	+	-
Z-38	7.17	33.7	11.8	-	-	-	-	-
Z-39	7.35	39.3	12.9	-	-	-	-	-
Z-40	7.65	37.9	13.1	-	-	-	+	-
Z-41	7.37	36	12.2	-	-	-	+	-
Z-42	5.61	29.1	8.9	-	+	-	+	-
Z-43	6.67	34.6	11.7	-	-	-	+	-
Z-44	6.03	29.1	10.3	-	-	-	+	-
Z-45	7.05	35.5	12.3	-	-	-	+	-
Z-46	8.15	36.6	13.5	-	+	-	+	-
Z-47	6.91	31.9	11.8	-	-	-	-	-
Z-48	8.45	41.2	14.5	-	-	-	+	-
Z-49	6.39	30	10.7	-	-	-	+	-
Z-50	7.73	34.9	13	-	-	-	+	-
Z-51	6.68	33.5	11.6	-	-	-	+	-
Z-52	5.43	26.4	9.2	-	-	-	+	-
Z-53	6.48	32.5	11.1	-	-	-	-	-
Z-54	8.46	39.8	14.4	-	+	-	-	-
Z-55	5.31	26.5	9.3	-	-	-	+	-
Z-56	4.74	22.8	8.5	-	-	-	+	-

RBC: red blood cells (normal range: 6.6– 10^6/μL); PCV: packed cell volume (normal range: 31%–50%); Hb: hemoglobin (normal range: 11.4–17.3 g/dL) [24, 25]; Abnormal: samples below normal range for at least one parameter. nPCR: nested PCR; cELISA: competitive ELISA. NA: not commercially available.

Supplementary Table 2. Ticks collected from horses in Nigeria.

Sample I.D	Tick species	Gender
2	*Amblyomma variegatum*	8 male
		8 female
4	*Rhipicephalus evertsi evertsi*	15 male
		20 female
6	*Rhipicephalus evertsi evertsi*	20 male
		30 female
7	*Amblyomma variegatum*	10 male
		3 female
	Rhipicephalus evertsi evertsi	9 male
		11 female
8	*Rhipicephalus evertsi evertsi*	6 male
9	*Boophilus decoloratus*	2 female
10	*Rhipicephalus evertsi evertsi*	15 male
		8 female
12	*Rhipicephalus sanguineus*	4 male
		6 female
14	*Rhipicephalus evertsi evertsi*	12 male
		8 female
15	*Amblyomma variegatum*	9 male
		14 female
16	*Rhipicephalus evertsi evertsi*	6 male
		1 male
	Boophilus decoloratus	2 female
17	*Rhipicephalus evertsi evertsi*	4 male
		7 female
18	*Rhipicephalus evertsi evertsi*	1 male
		1 female
20	*Hyalomma dromedarii*	7 female
	Hyalomma species	12 male
	Hyalomma impeltatum	4 female
26	*Rhipicephalus evertsi evertsi*	11 male
		10 female
29	*Rhipicephalus evertsi evertsi*	12 male
		4 female
Z-1	*Rhipicephalus evertsi evertsi*	2 male
		3 female
	Hyalomma species	1 male
		1 female
Z-2	*Rhipicephalus evertsi evertsi*	8 female
	Hyalomma truncatum	1 female
Z-3	*Boophilus decoloratus*	1 female
Z-4	*Rhipicephalus evertsi evertsi*	11 male
		10 female
Z-5	*Amblyomma variegatum*	10 male
		5 female<br>including 2 fully engorged female
Z-6	*Hyalomma dromedarii*	1 female
	Rhipicephalus evertsi evertsi	3 male
		1 female

Z-7	*Boophilus decoloratus*	2 female
	Rhipicephalus evertsi evertsi	6 male
		1 female
Z-8	*Rhipicephalus species*	2 male
	Hyalomma dromedarii	12 male
		1 female
Z-9	*Rhipicephalus evertsi evertsi*	18 male
		20 female
Z-10	*Hyalomma dromedarii*	10 male
		1 female
Z-11	*Rhipicephalus evertsi evertsi*	7 male
		11 female
Z-12	*Rhipicephalus species*	3 male
		1 female
Z-13	*Rhipicephalus evertsi evertsi*	6 male
		5 female
Z-14	No Ticks	
Z-15	No Ticks	
Z-16	No Ticks	
z-17	No Ticks	
Z-18	No Ticks	
Z-19	No Ticks	
Z-20	No Ticks	
Z-21	No Ticks	
Z-22	No Ticks	
Z-23	No Ticks	
Z-24	No Ticks	
Z-25	No Ticks	
Z-26	No Ticks	
Z-27	No Ticks	
Z-28	No Ticks	
Z-29	No Ticks	
Z-30	No Ticks	
Z-31	No Ticks	
Z-32	No Ticks	
Z-33	No Ticks	
Z-34	No Ticks	
Z-35	No Ticks	
Z-36	No Ticks	
Z-37	No Ticks	
Z-38	No Ticks	
Z-39	No Ticks	
Z-40	No Ticks	
Z-41	No Ticks	
Z-42	No Ticks	
Z-43	No Ticks	
Z-44	No Ticks	
Z-45	No Ticks	
Z-46	No Ticks	
Z-47	No Ticks	
Z-48	No Ticks	

Z-49	No Ticks	
Z-50	No Ticks	
Z-51	No Ticks	
Z-52	No Ticks	
Z-53	No Ticks	
Z-54	No Ticks	
Z-55	No Ticks	
Z-56	No Ticks	
